# Endocrine Modulation in Long-Term Karate Practitioners

**DOI:** 10.1155/2018/1074801

**Published:** 2018-10-16

**Authors:** Francisca M. Vera, Juan M. Manzaneque, Gabriel A. Carranque, Francisco M. Rodríguez-Peña, Soledad Sánchez-Montes, María J. Blanca

**Affiliations:** ^1^Universidad de Málaga, Andalucía Tech, Departamento de Psicobiología y Metodología de las CC. del Comportamiento, Campus de Teatinos s/n, 29071 Málaga, Spain; ^2^Universidad de Málaga, Andalucía Tech, Departamento de Cirugía, Campus de Teatinos s/n, 29010 Málaga, Spain; ^3^Unidad de Gestión Clínica de Laboratorio AGS Este de Málaga-Axarquía. Málaga, Spain

## Abstract

**Purpose:**

Karate is a martial arts discipline which is widely practiced in the Western world as a form of self-defense, as well as a discipline to achieve physical and mental balance. However, little is known with respect to its specific psychobiological effects, particularly in relation to the influence that it may exert on the endocrine system. Thus, in the present study, we examined the effects of karate on several hormonal parameters of the Hypothalamic-Pituitary-Adrenal and Hypothalamic-Pituitary-Thyroid axes in long-time practitioners.

**Methods:**

Twenty-two healthy volunteer subjects (12 experimental and 10 controls) participated in the study. Experimental subjects were karate players with a minimum of 3 years of practice in this discipline. Blood samples for the quantification of hormonal parameters were taken in both groups. The Mann-Whitney* U* test was performed for each variable in order to analyze the differences between groups.

**Results:**

Statistically significant differences were found in cortisol and thyroid hormones, with the karate group showing lower levels of these hormones as compared to control.

**Conclusions:**

These findings, therefore, reveal that long-term karate practice is associated with a significant endocrine modulation, which suggests interesting psychobiological and clinical implications. Further research is needed to verify these preliminary results, as well as properly assessing its possible use as a psychosomatic intervention tool.

## 1. Introduction 

Karate, literally translated as ‘empty hand' (*kara *meaning empty, and* te *meaning hand), is a Japanese martial art developed in Okinawa at the XIX and XX century. This discipline of martial arts is practiced for self-defense and to improve health [1] and represents one of the most popular combat sports around the world [2, 3]. Karate predominantly consists of striking techniques that involve the use of one's bare hands, elbows, feet, and knees [4]. Its training improves the cardiorespiratory fitness, increases muscle strength, muscle endurance, and flexibility, and promotes reaction capability [5, 6].

In general, martial arts are considered a form of exercise that focuses on the correct mental and physical balance. Traditional martial arts practice is not limited to teaching self-defense but involves philosophical and ethical teachings to be applied to life [7]. Likewise, a high degree of ceremony and ritual, emphasis on integration of mind and body, and a meditative component are elements included in their practices [8]. Karate, in particular, which is widely practiced in the Western world, has been proposed as a way to achieve a sense of self-mastery and self-regulation, motor and mental coordination, and inner harmony. This discipline combines physical activity with the cognitive training of focusing attention, coordinating one's thinking, as well as self-monitoring, having been described as a sort of “moving meditation” [9].

Scientific research has revealed that karate practice can exert beneficial effects on mental and physical health [e.g., [7, 9–13]]. However, very little is known with respect to its specific psychobiological effects, particularly in relation to the influence that it may exert on the endocrine system. Although the findings reported in literature are often contradictory, martial arts, in general, have been said to be a good model for investigating neuroendocrine responses to competitive fighting [14]. In this context, for example, a significant postsession increase of cortisol and catecholamine levels has been reported in mixed martial arts competitors [15] and in Marine Corps martial arts training [16], respectively. Similarly, Salvador [17] found increases of cortisol and testosterone in winning judo athletes, while reporting decreases of these parameters in losing judo competitors. Interestingly, these results contrast with those reported by Benedine et al. [14] in karate athletes, since they observed higher cortisol levels in losers than in winners. It is important to point out, however, that the aforementioned studies were carried out in the context of competitive fight and with distinct martial arts disciplines, which may account for the difference in results. Nevertheless, there is still a marked lack of scientific research about the possible hormonal modulation, beyond mere sport combat situations, derived from traditional karate practice.

Hypothalamic-Pituitary-Adrenal (HPA) axis function has been widely evaluated for its involvement on health, in general, and well-being and quality of life, in particular. Thus, a relationship between dysregulation of the levels of HPA axis hormones and pathologies associated with stress, including poor sleep quality, negative mood, anxiety, and depression has been reported [18–27].

It has been shown that the blood levels of adrenal hormone cortisol rise with exercise as a result from activation of the HPA axis [28]. In this sense, karate training, which involves a considerable physical activity, could produce a stimulation of this hormonal axis. In this respect, an increase on blood cortisol levels immediately after karate practice has been reported [3, 29]; likewise, no changes on the levels of this hormone have been observed [14]. These researches, however, did not explore the modulatory action of this martial art on other hormones of the HPA axis, like adrenocorticotropic hormone (ACTH) or dehydroepiandrosterone sulphate (DHEA-S).

On the other hand, alterations in thyroid hormones have been also linked to psychiatric diseases [30, 31]. The Hypothalamic-Pituitary-Thyroid (HPT) axis activity, with its widespread influence on physiology and behavior [32], can be affected by physical exercise like the HPA axis, decreasing or increasing thyroid hormone levels in response to exercise [33, 34]. In this sense, it has been proposed that the specific influence of exercise on thyroid function is controversial and seems to depend on the exercise type and the intensity and the duration of the training program [35, 36]. Regarding the possible influence of karate practice on HPT axis activity, there is a significant lack of scientific research; in this respect, we have found only one study, with another martial art discipline used, showing no significant changes on the levels of thyroid-stimulating hormone (TSH) and thyroxine (T4) [37].

Karate practice, like other martial arts, includes a relevant and integrative mind-body component which entails itself an interesting holistic approach. It seems logical, therefore, that the possible beneficial effects of karate on health occur by both, the physical and mental or psychological components; in this sense, researching the psychobiological influence of its training could provide relevant information about the possible implications in the clinical field and health. An interesting way to provide information about the psychobiological modulation of karate practice can be exploring its effects on neuroendocrine parameters.

Although karate has been demonstrated to exert a significant hormonal modulation, very few previous investigations have focused on cortisol with the aim of examining the acute endocrine effects of karate, immediately after practice. To the best of our knowledge, no study has ever investigated the potentially interesting action of regular karate practice on HPA axis activity, not limited to a punctual situation of fighting. In addition, no study has evaluated the possible modulatory action of this martial art discipline on HPT axis function, and the potential impact of long-term karate practice has not been explored yet. Therefore, our study may shed light on the modulatory action of karate on HPA and HPT axes function in long-term practitioners. Thus, the present study provides a first attempt towards exploring the neuroendocrine modulation of regular karate practice. Specifically, the purpose of this work has been to examine the effects of karate on several hormonal parameters of the HPA (ACTH, cortisol, and DHEA-S) and HPT axes (TSH, T3, and T4) in long-time practitioners.

## 2. Methods

### 2.1. Participants

Twenty-two healthy men, with ages ranging from 18 to 55, participated voluntarily in this study. The experimental or karate group consisted of 12 long-term karate practitioners, while the control group was made up by 10 ordinary subjects.

A medical and psychological interview (including the trait form of the Spielberger State-Trait Anxiety Inventory, STAI) was carried out for the selection of participants. Subjects were screened to exclude those with any pathological conditions and/or those who had followed pharmacological treatment in the last 3 months prior to the experiment. Only healthy men, with regular life habits, who did not take any type of drugs, were chosen to be part of the experimental or control groups. All subjects selected to be part of the control or experimental group were contacted in the following days after their initial interview.

Experimental subjects were regular karate players with a minimum of 3 years of practice in this discipline (M = 4.05, SD = 0.76). They were recruited from several karate training centers in Malaga province (where about 100 people practiced regularly) and selected from among a larger group of 25 volunteers, who decided to take part in the study, following an oral announcement in the training center. Only male participants were recruited, mainly, because it was by far the most accessible sample in the training centers, and actually, owing to this a balanced sex ratio in the experimental group would not have been possible. Control subjects were selected from 30 healthy candidates without disease or suspicion of disease that were programmed to go to hospital for a routine medical check-up. These subjects were therefore not in the hospital owing to any pathology that could have compromised the representativity of the control sample. These individuals were of the same age and sex as the experimental group and followed a similar lifestyle but had no experience on karate or similar practices. Age and lifestyle were the most difficult variables to match, and, at the end of the selection, only 10 control subjects matching the experimental group were available.

The study protocol was conducted according to the Declaration of Helsinki and was approved by the Institutional Ethical Committee. Informed consent was obtained from all individual participants.

Table 1 summarizes information on age, height, and weight of all participants, as well as anxiety scores (STAI). As it is shown, none of these variables were significantly different between the control and experimental groups.

### 2.2. Karate Intervention

Karate training in the experimental group typically entailed two to three lessons a week, with a duration of about one hour each. Experimental subjects had been following this routine for a minimum of 3 years.

The practice of karate traditionally consists of “kata” (a prearranged solo fighting sequence), combat drills (short fighting techniques practiced with a partner), and “kumite” (a sparring practice involving two partners). The main feature of traditional karate lies in the balance, in terms of importance and dedication, given to these three components. This is especially true in the particular style of karate practiced by the experimental subjects, namely, Shito Ryu.

### 2.3. Blood Sampling

Blood samples were taken from all subjects for the quantification of hormones of the Hypothalamic-Pituitary-Adrenal (HPA) axis, adrenocorticotropic hormone (ACTH), cortisol, and dehydroepiandrosterone sulphate (DHEA-S), and the Hypothalamic-Pituitary-Thyroid (HPT) axis, thyroid-stimulating hormone (TSH), free triiodothyronine (free-T3), and free thyroxine (free-T4). Experimental subjects gathered at the hospital (“Unidad de Gestión Clínica de Laboratorio” of AGS Este de Málaga-Axarquía) for a period of one week, coinciding with the appointment of control participants. Blood was drawn by venepuncture at 9:00 h.

ACTH was collected into tubes containing EDTA, centrifuged immediately in a refrigerated centrifuge, and stored at – 20°C. Plasma ACTH concentrations were analyzed with a two-site chemoluminescence immunometric assay.

TSH, free-T3, free-T4, cortisol, and DHEA-S levels were assessed with a competitive chemiluminescence enzyme immunoassay. All samples were quantified by using MODULAR ANALYTICS E170 (ROCHE DIAGNOSTICS).

### 2.4. Statistical Analyses

The Mann-Whitney* U* test was performed for each variable in order to analyze the differences between the control and karate groups and* r* was calculated as an effect size measure.

## 3. Results

Results from the Mann-Whitney* U* test are presented in Table 2. Differences were found in cortisol and thyroid hormones (free-T3 and free-T4), with the karate group showing a lower level. According to Cohen's criterion [38], the effect size values indicated a large difference in these variables. Likewise, regarding DHEA-S, the* U* test was not significant but it was near .05, with a medium effect size value.

## 4. Discussion

The main finding of the present investigation is that regular karate practice is associated with decreased blood levels of cortisol and thyroid hormones. Our data, therefore, show a significant endocrine modulation on several relevant components of the HPA and HPT axes. These results are, to the best of our knowledge, the first ones to be published to date employing long-time practitioners of this discipline of martial arts.

Our results in cortisol appear to suggest that karate practice is related to a decrease in the function of the HPA axis; however, no significant differences were observed in ACTH and DHEA-S. Nevertheless, a medium effect size value has been found on DHEA-S, another hormone released from the adrenal gland under the influence of ACTH like cortisol.

Given the very scarce scientific literature about the effect of long-term karate practice on HPA axis, we do not know of similar studies to ours that can allow us to establish appropriate comparisons. The very few existing studies evaluate the acute effects of karate on cortisol immediately after practice, and some of them have shown a significant increase on the levels of this hormone [3, 29]. However, curiously, in a previous study using yoga, a mind-body oriental discipline, we have observed, like in our research, an association between regular practice and modulation of the levels of cortisol, with no significant differences in ACTH and DHEA-S [39].

On the other hand, regarding our results on hormones of the HPT axis, we have found that regular karate practice appears to induce modulatory effects on the endogenous secretion of thyroid hormones; however, this endocrine influence does not seem to have affected the blood levels of the pituitary hormone, TSH. We can say that an association between long-term karate training and a decrease of the HPT axis function has been also observed like on the HPA axis.

Thyroid hormones are critical to normal physiological function throughout life [34, 40]. However, thyroid axis has received much less attention than hormones of the HPA axis, particularly regarding its involvement on stress regulation. It has been proposed that the HPA axis can exert a role on the HPT axis function on stress conditions; thus, it has been reported that glucocorticoids can inhibit the HPT axis activity [32, 41]. In our study, karate practitioners displayed lower blood levels on both cortisol and thyroid hormones. It is interesting to note that our participants were not subjected to any physical or emotional acute stress condition, unlike what is described on the two aforementioned studies; therefore, the design of our study does not permit the discussion of the results on the same context.

Importantly, lower levels of cortisol and thyroid hormones on karate practitioners are not associated with significant differences on the levels of their respective pituitary hormones, ACTH and TSH, which regulate their secretion. Therefore, given similar concentrations of pituitary hormones in both the experimental and control subjects, only the experimental group has displayed a downregulation of hormonal axes, through the reduction of their end product levels. In our opinion, endocrine modulation reflects, in regular karate practitioners, an interesting psychobiological effect.

It is well-known fact that the HPA and HPT axes contribute to the maintenance of homeostasis in the body through impact on a multitude of physiological systems [36, 42], their impact on physical and mental health having been reported [19, 42–46]. From this point of view, our results show that long-term karate practice appears to be associated with an interesting modulatory influence on relevant biological parameters related to health and well-being. Recently, it has been suggested that the HPA axis modulation by therapeutic interventions may have a role in disease treatment and prevention [47]. Given that an association between high cortisol levels and adverse health outcomes has been strongly documented [48–53], the lower cortisol levels observed in karateka subjects seem to invite us to glimpse some clinical implications, which suggest that karate practice could constitute an useful psychosomatic tool. Likewise, the lower levels of thyroid hormones also point to interesting psychobiological implications; more information to be able to interpret this endocrine action of karate in terms of effects on health is needed.

The small number of subjects employed in this preliminary study could be considered as a limitation of its overall results; regarding results of DHEA-S, it is possible that the sample size is not large enough to achieve statistically significant results. Nonetheless, this limitation may be, in several ways, intrinsic to the nature of this research. The recruitment of long-time karate practitioners, who trained on a regular basis without significant gaps, while at the same time matching the characteristics of controls, was the most difficult task in this research. Likewise, the fact that the endocrine parameters were assessed only once throughout the study may constitute, to a certain extent, another limitation. However, the long-term nature of the practice of the experimental subjects proved that it is difficult to be able to obtain more than one measure whose evolution could be compared after years. Further studies should indeed include a larger sample size, male and female subjects, more than one measure throughout time, and several measures per day. This would provide a more reliable, complete, and broader perspective of the long-time effects of karate and its influence on the endocrine secretion profile. Finally, for future research, it would be interesting to compare karate to other conditions with the same level of intensity (active control or more traditional fitness training, for instance), so as to gain more knowledge about the reach of the effects obtained.

## 5. Conclusions

The present work represents a first attempt to explore the impact of long-term karate practice on several hormones of the HPA and HPT axes. The endocrine modulation found in regular karate practitioners suggests which may have interesting psychobiological and clinical implications. Further research is needed to verify these preliminary results, as well as properly assess its possible clinical potential as a psychosomatic intervention tool.

## Figures and Tables

**Table 1:** Mean ± standard deviation of age, height, weight, and STAI-R score in the control and karate groups, as well as independent sample *t*-test and *p *value.

Variable	Control Group	Karate Group	*t*	*p*
Age (yr)	28.50 ± 10.45	36.83 ± 14.15	-1.54	.14
Height (cm)	179.83 ± 6.04	177.33 ± 8.72	0.63	.54
Weight (kg)	79.33 ± 9.33	80.33 ± 12.27	-0.18	.86
STAI-R (score)	15.00± 9.07	14.58 ± 13.64	0.08	.93

**Table 2:** Descriptive statistic for hormones, Mann-Whitney *U* statistic, one-tailed *p* value, and effect size (*r*).

	Control Group			Karate Group					
Hormone	Mean	SD	Median	Mean	SD	Median	*U*	*p*	*r*
ACTH (pg/ml)	30.64	23.76	20.10	32.42	15.10	28.50	44	.16	.26
Cortisol (*μ*g/dl)	17.55	6.04	17.44	14.04	2.96	14.00	33	.04	.45
DHEA (ng/ml)	3371.70	1233.98	3214.50	2543.50	1329.93	2287.50	37	.07	.38
TSH (*μ*UI/ml)	2.48	1.31	2.15	2.42	0.97	2.21	59	.49	.02
free-T4 (ng/dl)	1.45	0.13	1.45	1.23	0.13	1.23	17.5	<.01	.71
free-T3 (pg/ml)	3.72	0.37	3.81	3.42	0.23	3.42	23	.03	.62

## Data Availability

The data used to support the findings of this study are available from the corresponding author upon request.
